# Feasibility study of subject‐specific, brain specific‐absorption‐rate maps retrieved from MRI data

**DOI:** 10.1002/mrm.30547

**Published:** 2025-05-24

**Authors:** Jessica A. Martinez, Umberto Zanovello, Alessandro Arduino, Houchun Harry Hu, Kevin Moulin, Stephen E. Ogier, Oriano Bottauscio, Luca Zilberti, Kathryn E. Keenan

**Affiliations:** ^1^ Physical Measurement Laboratory National Institute of Standards and Technology Boulder Colorado USA; ^2^ Department of Physics University of Colorado Boulder Boulder Colorado USA; ^3^ Istituto Nazionale di Ricerca Metrologica Torino Italy; ^4^ Department of Radiology, Section of Radiological Science University of Colorado Denver, Anschutz Medical Campus Aurora Colorado USA; ^5^ Department of Cardiology of Boston Children's Hospital Harvard Medical School Boston Massachusetts USA

**Keywords:** EPT, RF heating, SAR

## Abstract

**Introduction:**

Specific absorption rate (SAR) is crucial for monitoring radiofrequency power absorption during MRI. Although local SAR distribution is usually calculated through numerical simulations, they are impractical during exams, limiting real‐time patient‐specific SAR assessment. This study confirms the feasibility of deriving in vivo, subject‐specific, image‐based SAR and 10‐g SAR maps directly from MRI data.

**Methods:**

Complex B_1_^+^ maps were derived by combining a B_1_^+^ product (XFL) magnitude sequence with balanced steady‐state free precession phase. Anatomical information and tissue masking were obtained from a T_1_ magnetization‐prepared rapid gradient echo sequence. Electrical conductivity maps were generated from balanced steady‐state free precession phase. Whole‐brain SAR maps were created from MRI data acquired at 3 T using a 32‐channel head coil on 2 healthy volunteers. A correction factor was applied to account for underestimation due to reliance on measurable B_1_^+^ data. Numerical simulations compared image‐based SAR with simulation‐based SAR distributions.

**Results:**

A multi‐slice image‐based brain SAR map was obtained in 12 min (9‐min acquisition, 3‐min SAR reconstruction). In vitro experiments validated B_1_^+^ distribution and electrical conductivity values. Calculated electrical conductivities for in vitro and in vivo experiments were within reference ranges. Image‐based SAR and 10‐g SAR maps showed a distribution similar to simulation‐based maps (*r* = 0.5) after correction.

**Conclusions:**

This study shows the feasibility of inline, subject‐specific SAR and 10‐g SAR maps from standard brain clinical sequences. Image‐based SAR maps can be a practical alternative during MRI exams when simulations are not feasible.

## 1 INTRODUCTION

The induced electric field (E‐field) from the radiofrequency (RF) magnetic field (B_1_) deposits RF power into the tissues under examination, leading to a temperature increase that may potentially damage tissue.^1^ The specific absorption rate (SAR, W/kg) measures the RF power absorbed by the tissue. Monitoring SAR is mandatory to avoid tissue damage for all patients, especially for vulnerable patients such as pediatric, elderly, and patients with implants.

To prevent potential burns, clinical MRI scanners operate under the so‐called normal operating mode SAR limits, which, for volume RF transmit coils, restrict the average whole‐body SAR to be at most 2 W/kg and the average head SAR to be at most 3.2 W/kg for neurological examinations.^2^ To ensure scanning under these limits, MRI scanners monitor the SAR average of the region under examination and adjust the B_1_ peak for a given sequence, considering the RF calibration and the patient's height and weight.^3^ SAR is proportional to the electrical conductivity and the square of the local electric‐field amplitude, which can create a deviation between the local area of examination (local SAR) and the SAR average. Field strength plays a role in the SAR distribution, given that RF inhomogeneity increases with field strength. The RF coil characteristics and configuration significantly affect the electromagnetic distribution, with multichannel transmit arrays presenting a more complex distribution than volume coils. The patient's build and posture, their position with respect to the RF coils, and the patient's tissue electrical properties (conductivity and permittivity) play a role in the local SAR distribution, as tissue heterogeneity and geometric complexity vary across the body. Thus, the average SAR value potentially overlooks regions within the patient, commonly known as hotspots, where the local SAR values could exceed safety limits, increasing the risk of burns.^4^, ^5^ Although the SAR safety standards^2^ provide local SAR limits for local coils, they do not account for volume coils, assuming that compliance with whole‐body SAR limits ensures safety. Subject‐specific local SAR assessments may offer more precise risk evaluations, reducing unnecessary safety margins and addressing the potential for local SAR hotspots even when whole‐body SAR limits are met.

Numerical simulations are commonly used to estimate SAR and hotspots.^6^, ^7^, ^8^, ^9^, ^10^, ^11^ Simulations enable the estimation of voxel‐wise, high‐resolution, electric‐field, and SAR maps over extensive body coverage. Simulations require detailed RF body coil characteristics, loading, and tuning conditions, in addition to the creation of a three‐dimensional (3D) model of the patient's body and the examined tissues' electrical properties values (conductivity and permittivity). The local SAR distribution for a given patient can be determined with voxel‐level precision using simulations that utilize tools for automatic tissue segmentation and 3D model generation,^12^ whereas the tissues' electrical properties can be estimated based on values reported in the literature.^13^, ^14^ However, numerical simulations are often computationally intensive; computational time can range from tens of minutes to hours, depending on the model's mesh resolution, the number of discretization cells, the use of GPU acceleration, and the type of solver used (FEM, FDTD).^15^ Recent work has aimed to address these computational limitations. For instance, fast electromagnetic simulation tools using accelerated integral equation methods have been developed to enable more efficient SAR calculations.^16^ These approaches nevertheless require detailed knowledge of the RF coil characteristics, including coil design configuration and RF port locations—information that remains challenging to obtain. Alternatively, for parallel transmit systems, deep learning approaches at 7 T have emerged for rapid SAR estimation by predicting SAR from measured B_1_^+^ maps^17^—another potential approach for reducing computational demands. However, they still require extensive training data, are still being validated for clinical implementation, and face challenges in handling patient‐specific variations in tissue properties and positioning. In addition, variations in tissue properties compared with the literature database can affect the SAR estimation, making local SAR assessment through simulations during an MRI exam impractical.

The limited information provided by the average SAR and the impracticality of performing numerical simulations during MRI scans mean that vulnerable patients often undergo MRI examinations without local SAR monitoring. Therefore, as per safety guidelines,^18^, ^19^ it is crucial to develop methods that allow the characterization of local SAR and hotspots during MRI exams using MRI data. The resulting maps must agree with those from numerical simulations, enabling the assessment of the patient's electrical properties and E‐field distribution within clinically feasible timeframes. This process involves characterizing the distribution of the complex B_1_ field, which allows for the retrieval of both the E‐field and the electrical properties within tissue.

The E‐field can be estimated by applying an approximation of Ampere's law to the complex B_1_^+^ field.^20^ Although no MRI sequence can simultaneously estimate the magnitude and phase of the B_1_^+^ field, the field can be approximated by combining the magnitude retrieved from a B_1_^+^ mapping method^21^, ^22^, ^23^, ^24^ with the phase from MR sequences with minimal B_0_ components.^20^ Although direct assessment of the B_1_^+^ field phase is not feasible, the transceive phase assumption suggests that, for circularly polarized fields, using a quadrature body coil for both transmission and reception with a polarization switch, or using a multichannel receiver coil with intensity non‐uniformity correction,^25^ the phase of sequences without significant B_0_ components can be approximated as twice the B_1_^+^ phase.^26^ Examples of such sequences include fast spin echo and balanced steady‐state free precession (bSSFP) sequences.^27^ Furthermore, tissue electrical properties can also be estimated using the B_1_^+^ field. Electrical properties tomography (EPT) is an emerging MR‐based method that uses the magnitude and the phase of the B_1_^+^ field to estimate electrical permittivity and conductivity.^26^, ^28^ Direct methods like Helmholtz‐EPT enable the rapid reconstruction of electrical conductivity.^28^

The use of the B_1_^+^ field to retrieve image‐based SAR maps has been previously proposed.^29^, ^30^, ^31^, ^32^, ^33^ This work aims to investigate the feasibility of acquiring in vivo image–based SAR maps that are equivalent to simulation‐based SAR maps in the brain and are suitable for clinical use within practical scan times. To achieve this, a sequence protocol and workflow are proposed to retrieve volumetric electrical conductivity, SAR, and SAR averaged over 10 g (10 g‐SAR) within 12 min. The retrieved image–based SAR maps are then compared with simulation‐based SAR maps.

## 2 METHODS

Data were acquired on a Siemens Skyra 3T scanner (Siemens Healthineers, Germany) with a 32‐channel head receiver coil for both in vitro and in vivo experiments. Image‐based SAR and 10‐g SAR maps were acquired in vivo, and the distribution was compared with simulation‐based SAR maps obtained through numerical simulations. In vitro comparison of image‐based and simulation‐based B_1_^+^ distribution was performed using a homogeneous spherical phantom, whereas electrical conductivity values were evaluated using a heterogeneous phantom with multiple saline concentrations.

### 2.1 MRI sequence protocol

All sequences implemented for electrical conductivity, B_1_^+^, and SAR maps are readily available for clinical use. A multislice two‐dimensional (2D) B_1_ mapping product (XFL^24^) sequence was used for obtaining B_1_^+^ magnitude, and a multislice 2D bSSFP sequence was used for acquiring the B_1_^+^ phase. The bSSFP phase was acquired with the Surface Coil Intensity Correction filter (Prescan Normalize) to ensure that bSSFP phase corresponds to twice the transmit phase.^25^ A T_1_‐weighted magnetization‐prepared rapid gradient echo (MPRAGE) sequence was used to obtain anatomical information for tissue segmentation and create a virtual model for electromagnetic simulations. The total scan time was 9 min and 17 s. Sequence parameters are summarized in Table 1. To ensure correct voxel positioning for SAR analysis, all acquired images were interpolated to match the orientation and resolution of the 2D bSSFP sequence using a custom‐made code (processing time = 28 s, Apple M3, 14 Cores, 4.05 GHz).

**TABLE 1:** MRI sequence parameters and implementation for the in vivo image‐based specific absorption rate maps.

	Sequence	Repetition time (ms)	Flip angle (°)	Echo time (ms)	Voxel size (mm)	Image size	Scan time (min:s)
Anatomical reference	MPRAGE^a^	2300	9	2.94	(1.2, 1.2, 1.2)	(192, 192, 208)	4:06
SAR analysis	bSSFP	200	25	2.36	(2.3, 2.3, 2.5)	(128, 128, 35)	4:54
	XFL	8190	8	1.97	(4, 5)	(64, 64, 30)	0:27

Abbreviations: bSSFP, balanced steady‐state free precession; MPRAGE, magnetization‐prepared gradient echo; SAR, specific absorption rate; XFL, B_1_^+^ product.

^a^ MPRAGE and the XFL sequence were interpolated to match the bSSFP parameters.

### 2.2 Simulation‐based SAR estimation

Simulation‐based SAR maps were retrieved using electromagnetic simulations. A high‐pass 3T (128 MHz) birdcage quadrature transmitter coil with characteristics similar to the scanner‐embedded birdcage coil was used to characterize ground‐truth simulation‐based B_1_^+^ and SAR distributions using the *Sim4Life* finite difference time domain (FDTD) solver (Zurich MedTech, Switzerland). The coil consisted of a 16‐leg body coil (diameter = 713 mm, height = 450 mm) with a cylindrical shield (diameter = 752 mm, height = 1500 mm). The coil conductors and shields were simulated as perfect electric conductors. The coil was tuned to the Larmor frequency using the ASTM phantom as a reference load. The coils were supplied via two ports spaced 90° apart in standard circular polarization mode. All simulation‐based maps were interpolated to match the orientation and resolution of the 2D bSSFP sequence. All simulations were performed using only head models truncated at the neck.

### 2.3 Electrical conductivity based on EPT

Electrical conductivity was retrieved using a phase‐based Helmholtz‐EPT approach with EPTlib v0.4.0^34^ applying a cuboid‐shaped Savitzky–Golay filter of size (5) to the bSSFP phase. To anatomically adapt the filter, the T_1_ MPRAGE images were used as reference with a weight parameter of 0.05. Additionally, the EPTlib postprocessing filter was applied using the reference image and a cubic‐shaped filter of size (10) with weight parameter of 0.1.

### 2.4 Image‐based SAR estimation

**FIGURE 1:**
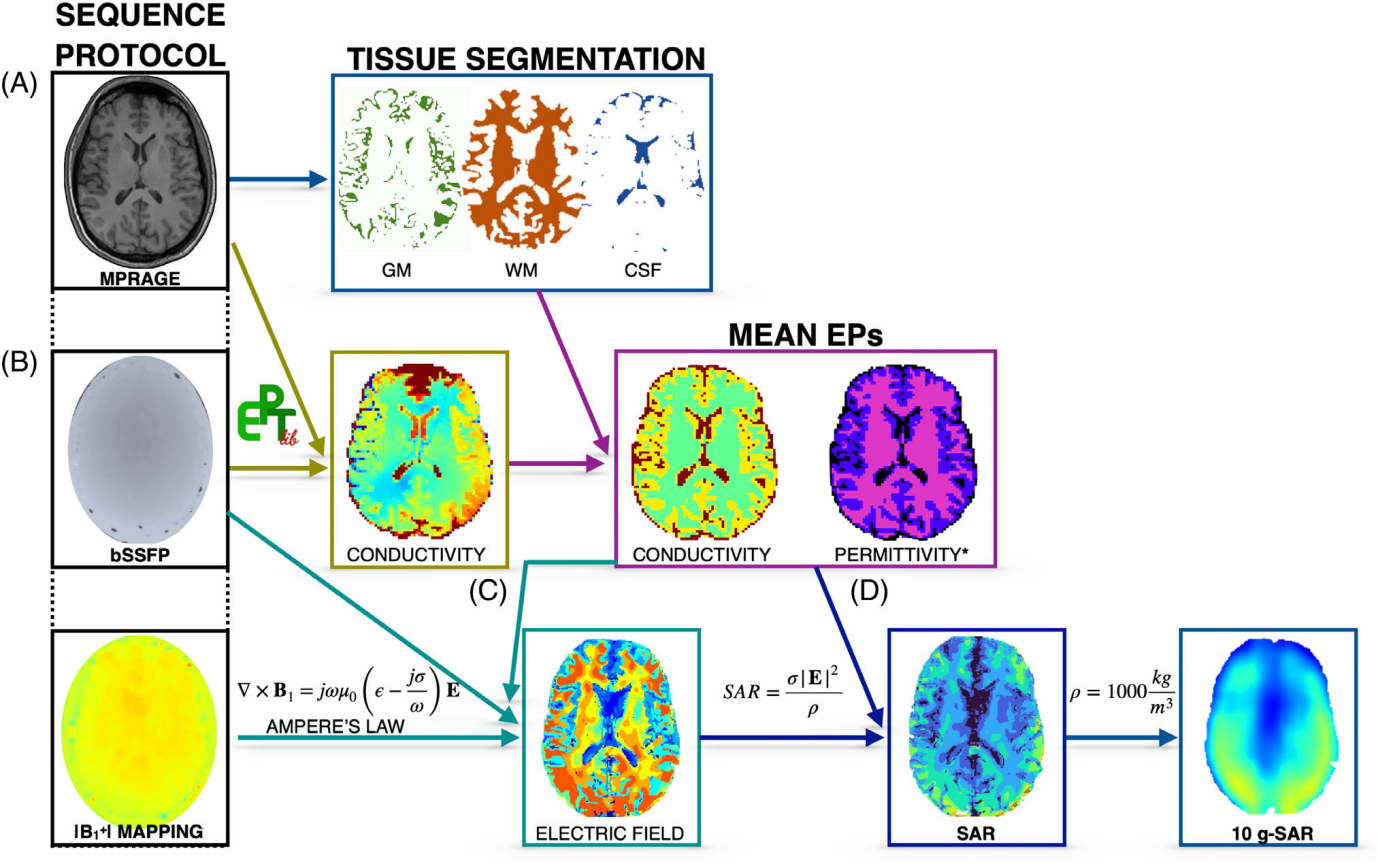
Workflow for deriving image‐based, subject‐specific specific absorption rate (SAR) maps. (A) A magnetization‐prepared rapid gradient echo (MPRAGE) sequence was used as an anatomical reference due to its high tissue contrast. (B) The phase of a balanced steady‐state free precession (bSSFP) sequence was used to retrieve electrical conductivity using a phase‐based Helmholtz–electrical properties tomography (EPT) approach. Mean electrical properties (conductivity and permittivity) maps per tissue were used to mitigate noise in subsequent steps. (C) The electric field was derived from the complex B_1_ field, constructed from the bSSFP and a B_1_^+^ magnitude sequence (in this case, B_1_^+^ product [XFL]) data along with the mean electrical properties. (D) SAR and 10‐g SAR were calculated assuming a tissue density of 1000 kg/m^3^. *The permittivity was obtained from literature sources. EP, electrical property.

Image‐based SAR maps were calculated using the B_1_^+^ information retrieved from MRI. Figure 1 illustrates the workflow for estimating image‐based SAR maps. First, the T_1_ MPRAGE was used for skull stripping and segmentation of white matter, gray matter, and cerebrospinal fluid (CSF) tissues using FSL.^35^, ^36^, ^37^ Second, electrical conductivity was obtained via a phase‐based Helmholtz‐EPT approach, and the mean values per segmented tissue were used to create a piecewise constant conductivity map to reduce noise artifacts. Third, the E‐field was approximated according to Ampere's law computing the derivatives of B_1_^+^ with an anatomically adapted Savitzky–Golay filter with ellipsoid shape and filter size of (4) and using the mean EPT electrical conductivity map and the electrical permittivity map from literature.^14^ Finally, the image‐based SAR map was calculated assuming a tissue density of 1000 kg/m^3^. A Gaussian filter (σ: 0.4) was applied to minimize overestimation due to noise propagation, followed by outlier removal. The Gaussian filter parameters were determined through empirical testing to reduce noise while preserving the SAR distribution features. The outlier removal threshold of mean + 9 standard deviations (SDs) was chosen based on statistical analysis to eliminate extreme artifacts while retaining legitimate high SAR values that could indicate actual hotspots. The code for SAR mapping is publicly available at https://github.com/DrJAMM/Image_Based_Subject_Specific_SAR. The total processing time was 3 min and 47 s (Windows virtual environment on Apple M3 processor, 4 CPUs, 4.05 GHz).

SAR maps calculated using only the B_1_^+^ component and excluding the B_1_^−^ and B_1z_ result in an underestimation; to correct for this, a tissue‐specific correction factor was applied. This factor, which represents the ratio between SAR calculated with the complete E‐field and SAR calculated with the E‐field derived from B_1_^+^, was determined using an extensive set of anatomical models.^33^ For the image‐based SAR maps, the correction factors were 3.08 for gray matter, 1.79 for white matter, and 2.59 for CSF.

### 2.5 10‐g SAR estimation

Local heating within a small tissue volume can be estimated using 10‐g SAR.^38^ To create these maps following the IEC 60601‐2‐33:2022 standard and ICNIRP Guidelines,^2^, ^39^ the image‐based and simulation‐based SAR maps were averaged over 10‐g cubes, assuming a tissue density of 1000 kg/m^3^, and computed using a custom *MATLAB* program. When approaching the tissue–air boundary, the cube volume was adjusted to include only tissue until the necessary 10‐g mass was achieved. Similarly to the image‐based SAR maps, the underestimation observed in the image‐based 10‐g SAR maps was corrected using a 10‐g SAR tissue‐specific correction factor.^33^ The correction factors were calculated using the 10‐g SAR derived from the whole E‐field and the 10‐g SAR derived from the E‐field calculated using only B_1_^+^ in a data set of simulated cases involving different anatomies and electrical properties.^33^ The retrieved correction factors were 2.11 for gray matter, 2.06 for white matter, and 1.95 for CSF.

### 2.6 Data acquisition

#### 2.6.1 In vitro phantom analysis

Before the SAR estimation, the image‐based B_1_^+^ mapping information and phase‐based Helmholtz‐EPT electrical conductivity values were compared with reference values in two phantom experiments.

The magnitude of the image‐based B_1_^+^ maps was compared with simulation‐based B_1_^+^ maps using a homogeneous Siemens Spherical Phantom D165 (10496625).^40^ A spherical phantom with similar characteristics to the imaged phantom was modeled in *Sim4Life* with the electromagnetic properties of distilled water at room temperature (20°C) and a frequency of 128 MHz (electrical conductivity = 0.01 S/m, relative electrical permittivity = 80).

A six‐compartment water‐based phantom was constructed for electrical conductivity mapping analysis. Each compartment consisted of 50‐mL conical vials filled with different concentrations of saline solution (NaCl) to achieve electrical conductivity values ranging from approximately 0.1 S/m to 0.7 S/m at room temperature (20°C).^41^, ^42^ The vials were arranged in a 1350‐mL cylindrical container filled with distilled water. Image‐based conductivity maps were compared with measurements using a dielectric assessment kit probe (DAK; SPEAG, Zürich, Switzerland) at room temperature (20°C) and a frequency of 128 MHz.

#### 2.6.2 In silico analysis

To validate the proposed image‐based SAR reconstruction method with a known ground truth, B_1_^+^ maps on the Duke virtual model were simulated and used for Helmholtz‐EPT conductivity reconstruction and SAR estimation. The model's tissue voxel map provided white matter, gray matter, and CSF tissue segmentation and served as the anatomical filter in both EPT and SAR analyses. The reconstructed conductivity and B_1_^+^‐based SAR maps were compared with the simulated SAR maps obtained with the *full* B_1_ field. For the in vivo generated models, the simulated B_1_^+^ field was used as an input to the image‐based methodology pipeline to compare B_1_^+^‐based SAR maps with the simulation‐based SAR maps obtained with the *full* B_1_ vector.

#### 2.6.3 In vivo analysis

Whole‐brain coverage 2D images were acquired for 2 healthy volunteers (1 female, 34 years, 54 kg; and 1 male 45 years, 77 kg) with informed consent under protocol number 02–729 approved by COMIRB ethics board. The image‐based SAR maps were obtained following the workflow summarized in Figure 1 and compared with simulation‐based SAR maps. A subject‐specific anatomical head model was generated using *Sim4Life* surface generation and automatic tissue segmentation from the T_1_ MPRAGE images (total model generation time = 18 min). For the simulation‐based SAR maps, the tissues other than white matter, gray matter, and CSF were assigned the tissue properties of fat.

#### 2.6.4 Data analysis

A mean absolute percentage error (MAPE) analysis was performed to compare the B_1_^+^ magnitude between the image‐based and the simulation‐based maps. The comparison was performed by dividing the maps into concentric regions (center, middle, outer) to assess spatial variations in agreement per slice. Correlation coefficients were also calculated. Both image‐based and simulation‐based SAR maps were normalized to their maximum values after Z‐score outlier removal (values >9 SD from mean). This normalization was only for comparative analysis This approach avoids sensitivity to local variations while comparing SAR patterns. To compare the values obtained with the image‐based method with those from the simulation‐based method, mean and SD values were calculated for both in vitro and in vivo experiments. Furthermore, for the in vivo analysis, a Pearson correlation was performed to assess the correlation between image‐based and simulation‐based SAR and 10‐g SAR value. A threshold of *p* < 0.05 was used to determine statistical significance.

## 3 RESULTS

### 3.1 In vitro phantom analysis

**FIGURE 2:**
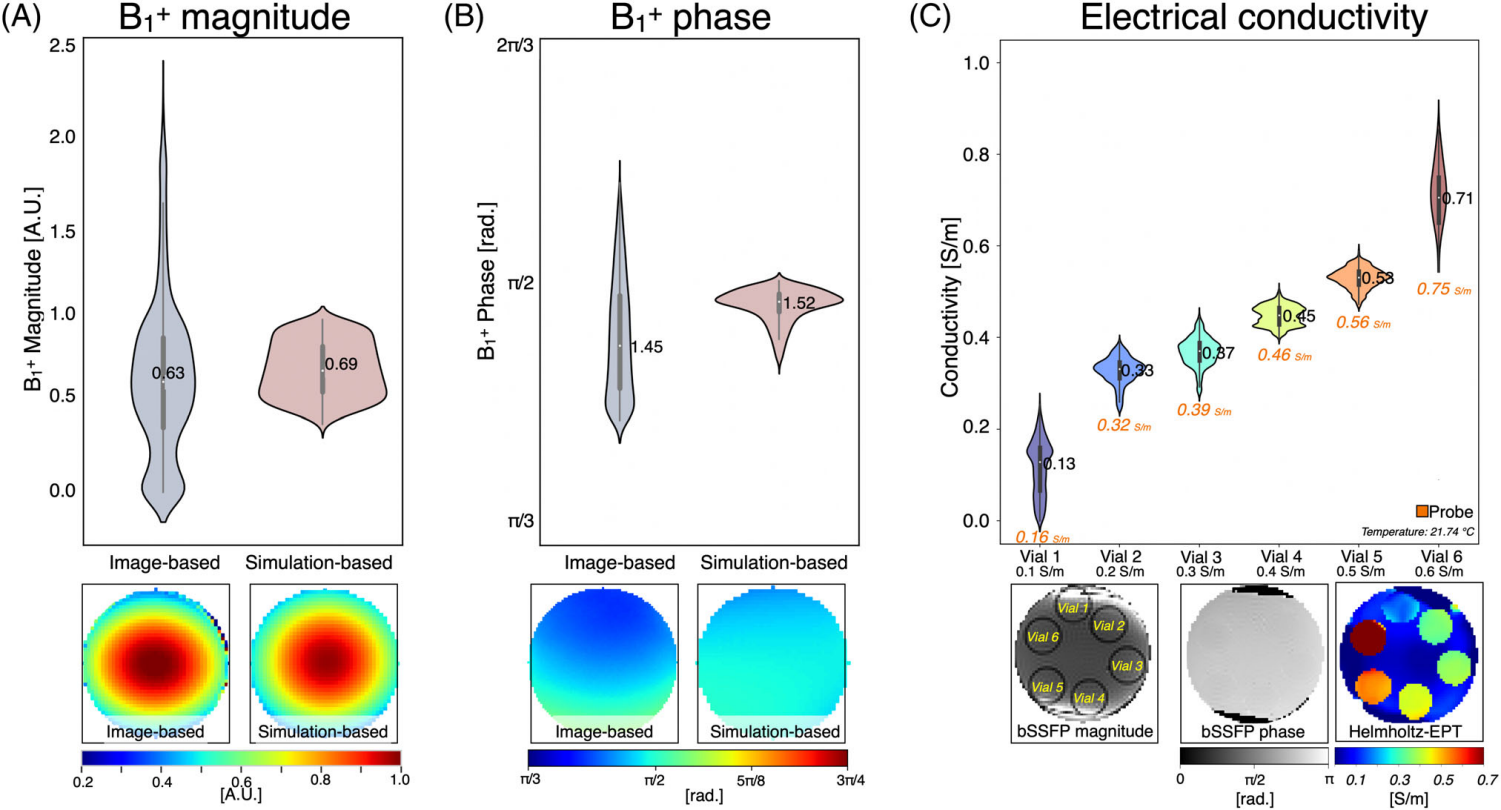
In vitro B_1_^+^ magnitude (A) and phase (B) distribution plots for the image‐based and simulation‐based method. (C) Electrical conductivity distribution plot per vial within the region of interest (*orange denotes values obtained with a dielectric probe kit*). Qualitative maps are shown below the plots.

Figure 2A,B displays the B_1_^+^ magnitude and phase distribution plots for both the image‐based and simulation‐based methods applied to the spherical water phantom. Qualitative B_1_^+^ maps are also presented. The image‐based maps show a broader spread of B_1_^+^ values compared with the simulation‐based maps. This spread may be due to noise and artifacts in the image‐based acquisition or due to limitations in the simulation, such as inaccuracies in the RF source's geometry, position, or supply. Despite this, the mean [SD] magnitude values were 0.63 [0.8] a.u. for the image‐based method and 0.69 [0.3] a.u. for the simulation‐based method, with a bias of +0.06 a.u. The mean [SD] phase values were 1.45 [0.13] rad for the image‐based method and 1.52 [0.04] rad for the simulation‐based method, with a bias of +0.07 rad.

Figure 2 c displays the distribution plot of the image‐based electrical conductivity obtained using the phase‐based Helmholtz‐EPT approach for each vial, along with the labeled bSSFP magnitude and phase images. The mean reconstructed conductivity values showed good agreement with probe measurements. The measured values were Vial 1 = 0.13 [0.06] S/m (probe: 0.16 S/m), Vial 2 = 0.33 [0.07] S/m (probe: 0.32 S/m), Vial 3 = 0.37 [0.08] S/m (probe: 0.39 S/m), Vial 4 = 0.45 [0.09] S/m (probe: 0.46 S/m), Vial 5 = 0.53 [0.09] S/m (probe: 0.56 S/m), and Vial 6 = 0.71 [0.11] S/m (probe: 0.75 S/m). The differences between reconstructed and probe measurements remained below 0.04 S/m for all vials. The SDs ranged from [0.06–0.11] S/m across the measured range.

### 3.2 In silico analysis

**FIGURE 3:**
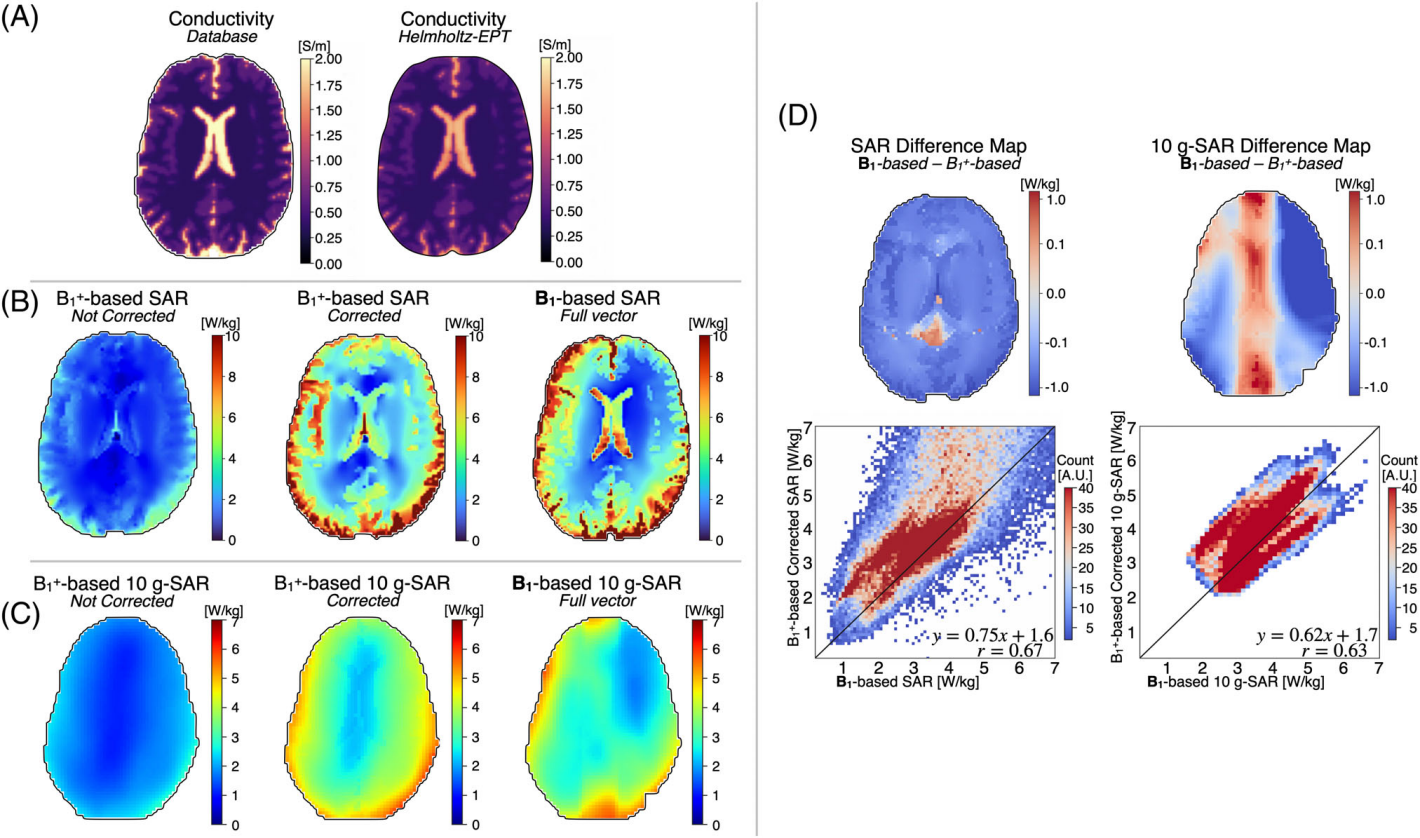
(A) In silico virtual model database (ground‐truth) and Helmholtz–electrical properties tomography (EPT) conductivity reconstruction from simulated B_1_^+^. (B) Specific absorption rate (SAR) maps obtained from the simulated B_1_^+^ and ground‐truth simulated SAR maps. The B_1_^+^ SAR maps were corrected using a correction factor. (C) 10‐g SAR maps obtained from the simulated B_1_^+^, and ground‐truth 10‐g SAR maps derived from the simulated SAR maps. (D) Difference SAR and 10‐g SAR maps and two‐dimensional voxel wise heatmap for the B_1_^+^ corrected and the simulation‐based method.

Figure 3 a shows single‐slice reconstructed conductivity maps. The mean [SD] reconstructed electrical conductivity was 0.58 [0.11] S/m for gray matter, 0.36 [0.08] S/m for white matter, and 1.69 [0.45] S/m for CSF. In comparison, the database values were 0.59 S/m for gray matter, 0.34 S/m for white matter, and 2.14 S/m for CSF. The reconstructed values show close agreement with the database, although a slight underestimation is observed in CSF (1.69 S/m vs. 2.14 S/m).

Figure 3 b,c present the single‐slice SAR and 10‐g SAR maps reconstructed from the simulated B_1_^+^ maps, both before and after applying the correction factor, alongside the *full vector* B_1_‐based SAR map. Figure 3 d displays the difference map between the B_1_‐based and corrected SAR and 10‐g SAR maps, as well as a 2D heatmap. Overall, the B_1_^+^‐based corrected SAR values were slightly underestimated compared with the B_1_‐based SAR, particularly in the CSF region. Nonetheless, the correlation coefficient *r* = 0.67 and *r* = 0.63 between the B_1_^+^‐corrected and the B_1_‐based SAR and 10‐g SAR, respectively, indicate a moderate positive linear relationship.

### 3.3 In vivo analysis

**FIGURE 4:**
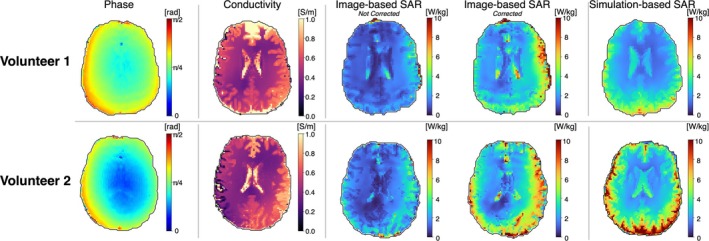
In vivo singe central slice qualitative image‐based b_1_^+^ phase, Helmholtz–electrical properties tomography (EPT) electrical conductivity, non‐corrected and corrected image‐based specific absorption rate (SAR) maps, and simulation‐based SAR map for the healthy volunteers. SAR maps were calculated using the Helmholtz‐EPT electrical conductivity. Image‐based SAR maps present a similar distribution compared with the simulation‐based SAR maps.

Figure 4 shows single‐slice maps for the 2 volunteers, including image‐based B_1_^+^ phase, image‐based Helmholtz‐EPT electrical conductivity, uncorrected and corrected image‐based SAR, and simulation‐based SAR. The mean [SD] values of Helmholtz‐EPT electrical conductivity for gray matter were 0.89 [0.35] S/m (Volunteer 1) and 0.55 [0.22] S/m (Volunteer 2); for white matter, they were 0.49 [0.12] S/m (Volunteer 1) and 0.44 [0.16] S/m (Volunteer 2); and for CSF, they were 1.63 [0.89] S/m (Volunteer 1) and 1.67 [0.7] S/m (Volunteer 2). In contrast, the electrical conductivity values from the database are 0.59 S/m for gray matter, 0.34 S/m for white matter, and 2.14 S/M for CSF. Regarding the SAR maps, both the uncorrected and corrected image‐based SAR maps align closely with the simulation‐based SAR maps for both volunteers. Certain regions such as near the ventricles or the anterior portion of the brain differ from the simulation‐based SAR maps, likely due to noise amplification, image artifacts, or inaccuracies in the E‐field estimation process, where signal contamination across tissue boundaries can occur that may not be corrected despite the use of the anatomical filter.

**FIGURE 5:**
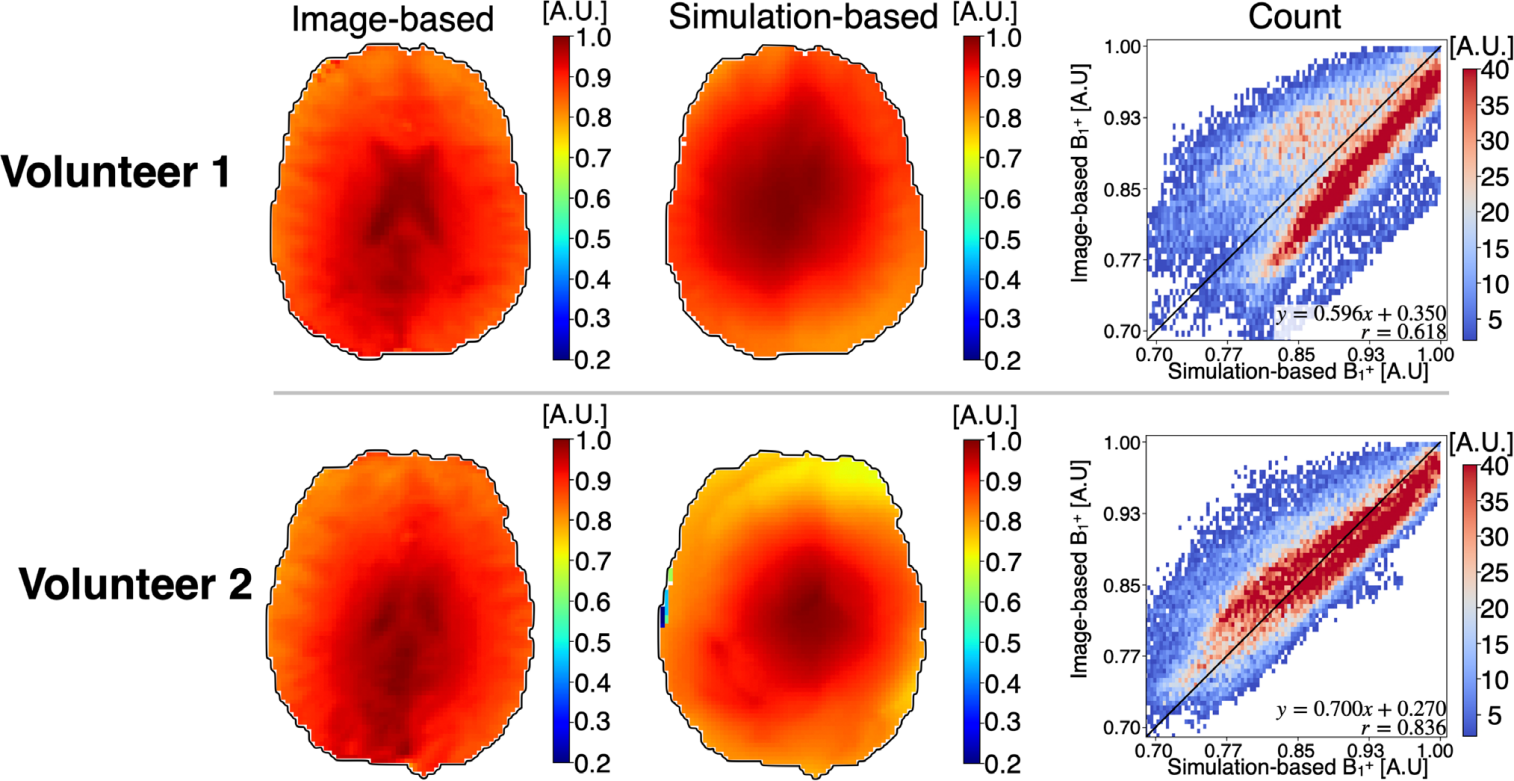
Single‐slice b_1_^+^ magnitude maps obtained from image‐based and simulation‐based methods for both volunteers, alongside two‐dimensional heatmaps comparing the two methods on a voxel‐wise basis.

Figure 5 shows single‐slice B_1_^+^ magnitude maps obtained from image‐based and simulation‐based methods for both volunteers, alongside 2D heatmaps comparing the two methods on a voxel‐wise basis. Both volunteers exhibited the best agreement in central regions (Volunteer 1: MAPE = 3.04%, Volunteer 2: MAPE = 2.48%), with agreement gradually decreasing toward the outer regions (Volunteer 1: MAPE = 6.63%, Volunteer 2: MAPE = 5.16%). Volunteer 2 achieved better overall correlation (*r* = 0.836 vs. *r* = 0.618) and lower global MAPE (3.83% vs. 5.71%) compared with Volunteer 1, reflecting intersubject variability in B_1_^+^ mapping accuracy.

**FIGURE 6:**
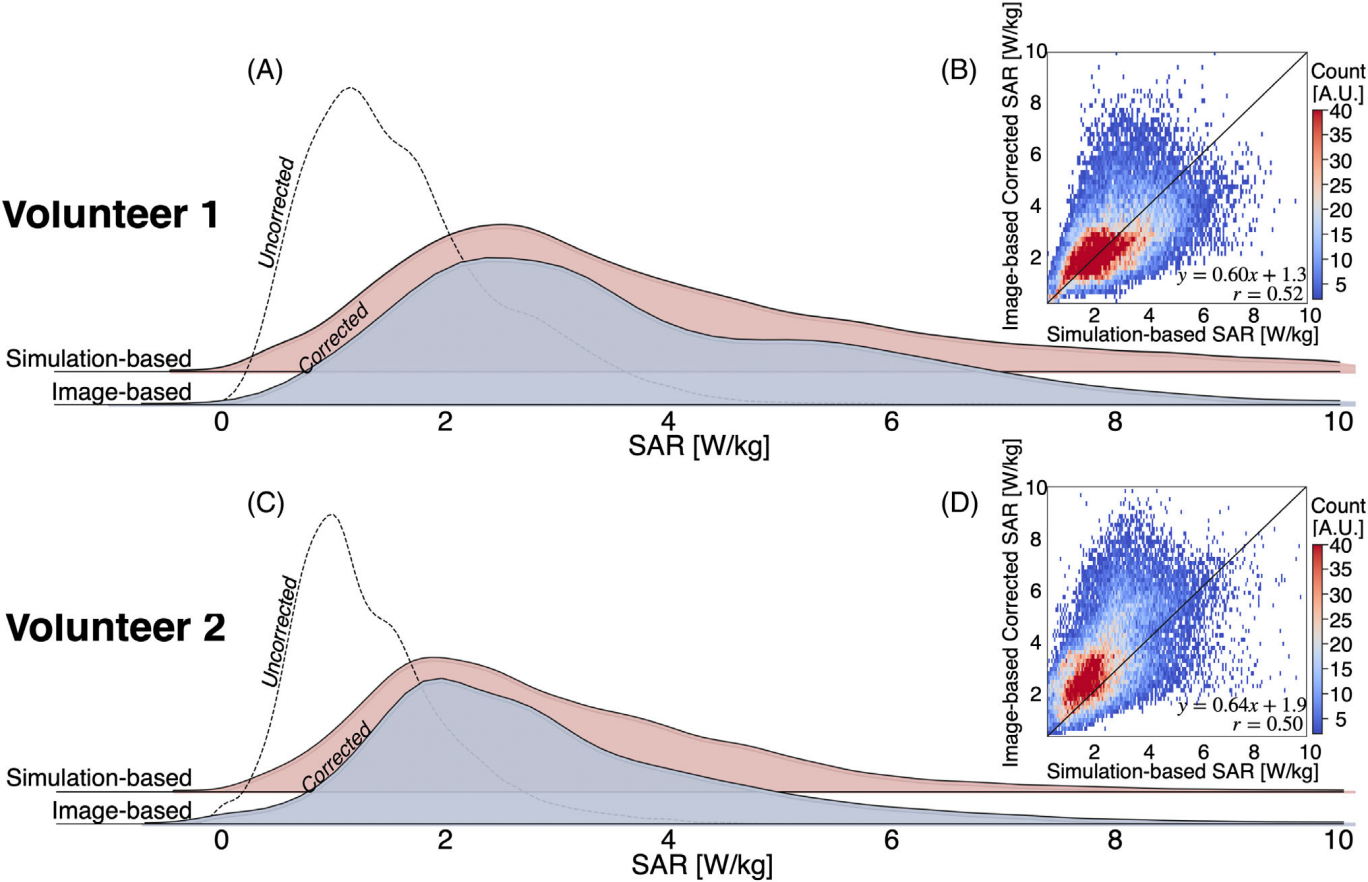
(A,C) Distribution plots for the image‐based and simulation‐based specific absorption rate (SAR) maps. Corrected image‐based SAR maps align more closely with the distribution of the simulation‐based SAR maps than the uncorrected image‐based SAR maps. (B,D) Two‐dimensional voxel‐wise heatmap for the image‐based corrected SAR versus simulation‐based SAR. Median [interquartile range] values for the uncorrected image–based, corrected image–based, and simulation‐based SAR maps were 1.2 [0.8] W/kg, 2.5 [1.9] W/kg, and 2.6 [1.0] W/kg for Volunteer 1, and 1.1 [0.7] W/kg, 2.3 [1.7] W/kg, and 2.2 [1.5] W/kg for Volunteer 2, respectively.

Figure 6 a,c presents distribution plots for the image‐based and simulation‐based SAR. For both volunteers, corrected image‐based SAR maps align more closely with the distribution of the simulation‐based SAR maps than the uncorrected image‐based SAR maps. Both the image‐based and simulation‐based SAR distributions are skewed to the right, indicating a nonnormal distribution. The skewness of the corrected image‐based SAR is significantly higher compared with the simulation‐based SAR for Volunteer 1 (4.3 vs. 1.0) and Volunteer 2 (2.1 vs. 1.2). Although this difference may be partially attributed to noise amplification during SAR computation, other factors such as B_1_^+^‐field inhomogeneities and tissue interface effects could also contribute to the observed skewness. Nonetheless, the median [interquartile range] values for the corrected image‐based and simulation‐based SAR maps were similar, with 2.5 [1.9] W/kg and 2.6 [1.0] W/kg for Volunteer 1, and 2.3 [1.7] W/kg and 2.2 [1.5] W/kg for Volunteer 2, respectively. In contrast, the uncorrected image‐based SAR maps had median [interquartile range] values of 1.2 [0.8] W/kg for Volunteer 1 and 1.1 [0.7] W/kg for Volunteer 2. Figure 6 b,d features a 2D heatmap comparing the corrected SAR to the simulation‐based SAR in a voxel‐wise manner. Although image artifacts and noise affected the reconstruction of the image‐based SAR, a Pearson correlation coefficient of *r* = 0.52 for Volunteer 1 and *r* = 0.50 for Volunteer 2 indicate a moderate positive correlation between the corrected image‐based SAR and the simulation‐based SAR, suggesting reasonable agreement between the two methods (*p* < 0.01).

**FIGURE 7:**
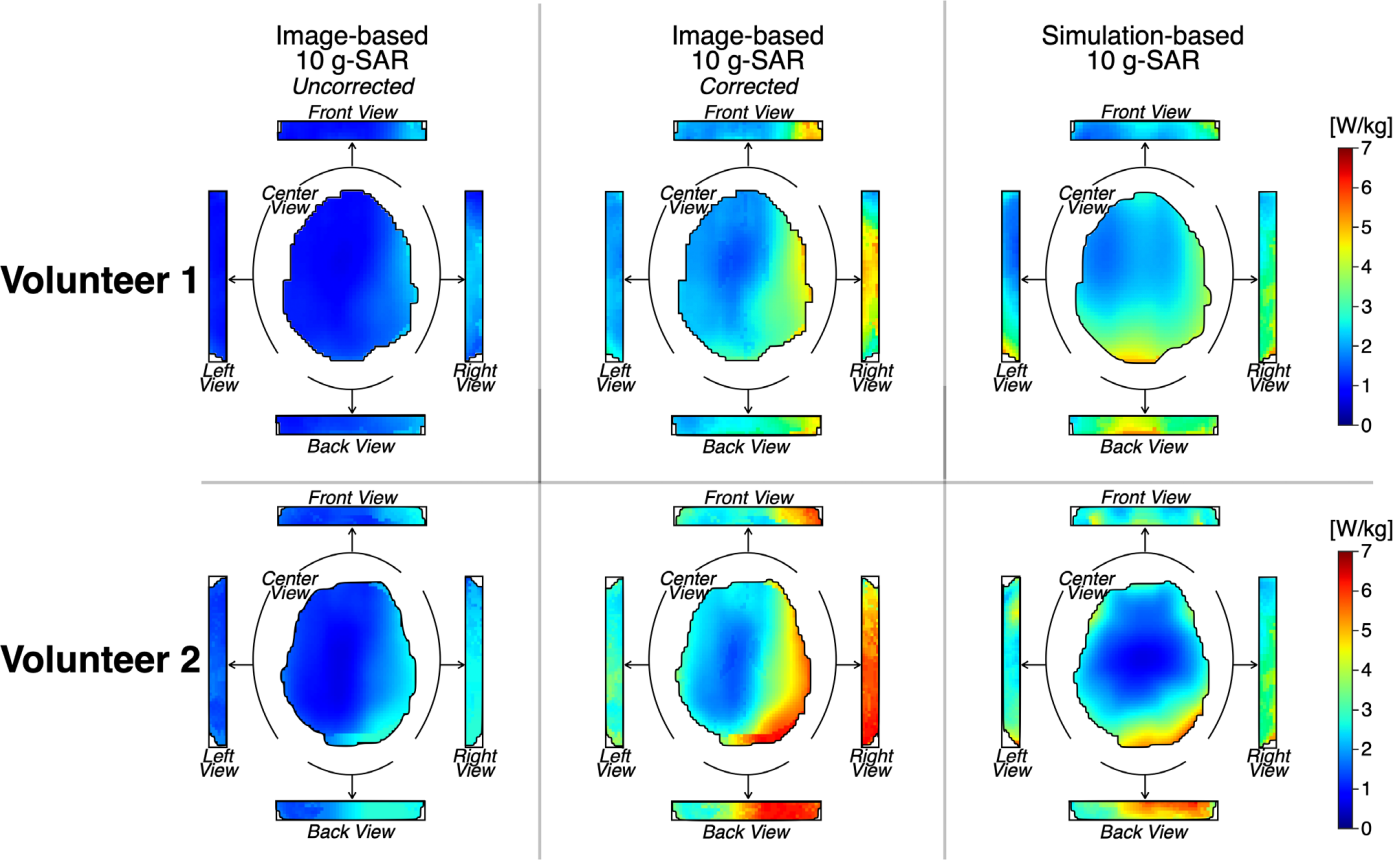
The 10‐g specific absorption rate (SAR) maps and projections in the center, front, back, right, and left views for the uncorrected and corrected image‐based and the simulation‐based methods for the healthy volunteers.

Figure 7 shows the 10‐g SAR maps and their surface value projections in the center, back, right, and left views for the uncorrected and corrected image‐based, as well as the simulation‐based, SAR maps. For both volunteers, the corrected image‐based maps demonstrate greater 10‐g SAR values in the right and posterior regions of the brain. Similarly, the simulation‐based maps also show high values in these regions, although the highest values were observed in the posterior part of the brain.

**FIGURE 8:**
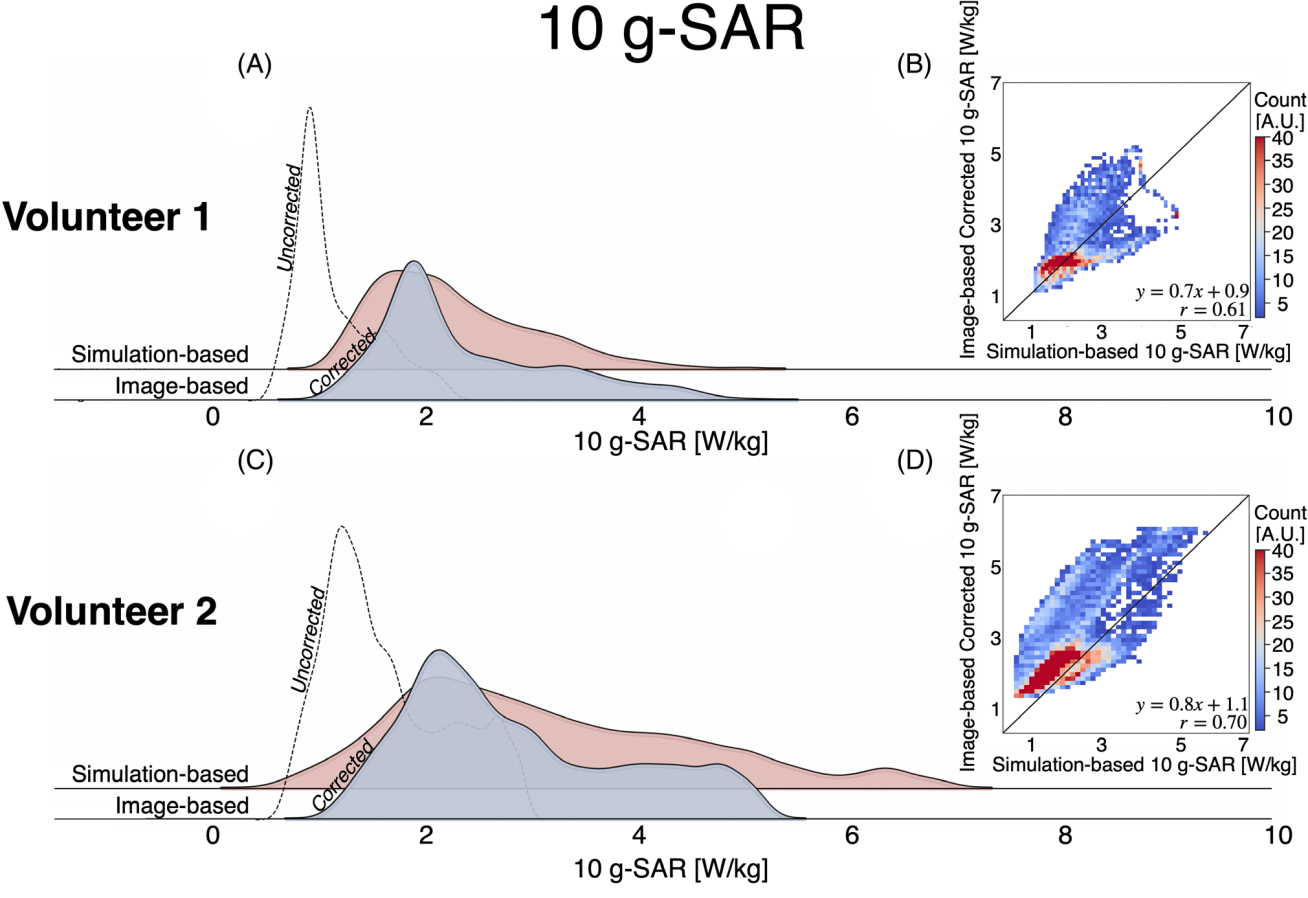
(A,C) Distribution plot for the image‐based and simulation‐based 10‐g specific absorption rate (SAR) maps. (B,D) Two‐dimensional voxel wise heatmap for the image‐based corrected 10‐g SAR versus simulation 10‐g SAR. Peak 10‐g SAR values were 5.22 W/kg (image‐based) and 5.04 W/kg (simulation‐based) for Volunteer 1 and 6.11 W/kg (image‐based) and 6.15 W/kg (simulation‐based) for Volunteer 2.

Figure 8 a,c presents the 10‐g SAR distribution plots for the image‐based and simulation‐based maps. Like the SAR maps, the corrected image‐based 10‐g SAR maps align more closely with the distribution of the simulation‐based 10‐g SAR maps than the uncorrected image‐based 10‐g SAR maps. The skewness of the corrected image‐based 10‐g SAR is 0.9, compared with 0.8 for the simulation‐based SAR in Volunteer 1, and 0.97 and 0.90, respectively, in Volunteer 2. Nonetheless, the median [interquartile range] values for the corrected image‐based and simulation‐based 10‐g SAR maps were similar (Volunteer 1: simulation‐based = 2.1 [1.0] W/kg, image‐based = 2.1 [1.1] W/kg; Volunteer 2: simulation‐based = 2.1 [1.3] W/kg, image‐based = 2.6 [1.6] W/kg). Figure 8 b,d presents a 2D heatmap comparing voxel‐wise the corrected 10‐g SAR to the simulation‐based 10‐g SAR. Overall, the distributions showed a moderate correlation (*r* = 0.61 and *r* = 0.70). Although the 10‐g SAR maps were strongly correlated in the 0–2‐W/kg range, the correlation weakened for higher SAR values, suggesting a birdcage loading conditions discrepancy between the image‐based and simulation‐based SAR maps that affected the location of the 10‐g SAR hotspot location. Nevertheless, both methods were significantly correlated (*p* < 0.05). For Volunteer 1, the peak 10‐g SAR values were 5.22 W/kg (image‐based) and 5.04 W/kg (simulation‐based), whereas for Volunteer 2, the peak 10‐g SAR values were 6.11 W/kg (image‐based) and 6.15 W/kg (simulation‐based).

**FIGURE 9:**
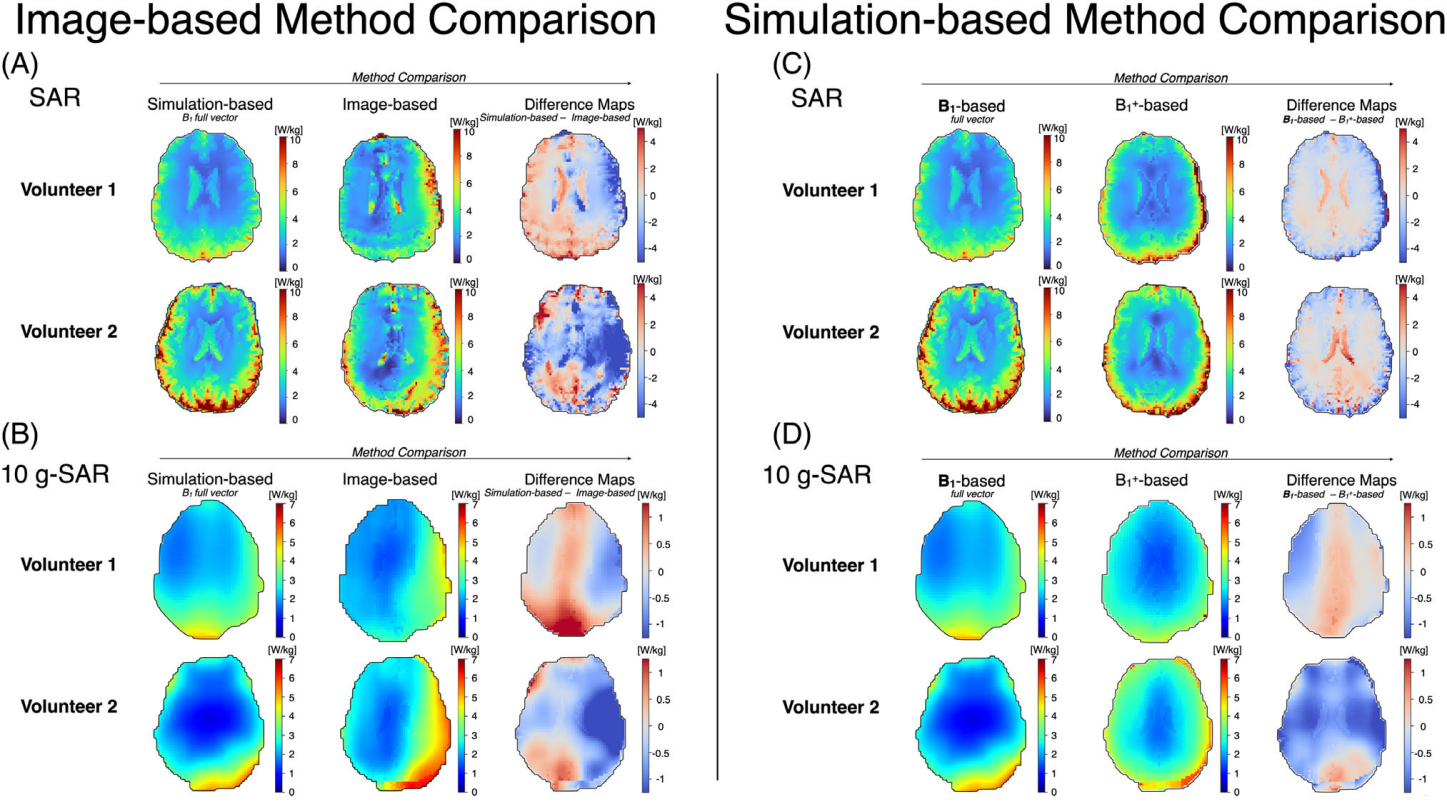
Method comparisons for specific absorption rate (SAR) and 10‐g SAR mapping. (A,B) Image‐based method comparison: simulation‐based versus MRI image–based SAR (A) and 10‐g SAR (B) maps. (C,D) Simulation‐based method comparison: complete B_1_ field versus B_1_^+^‐only simulations for SAR (C) and 10‐g SAR (D) maps. The simulation‐based approach uses the complete B_1_ field (including magnitude and phase information), whereas the image‐based method (A,B) uses B_1_^+^ values retrieved from MRI data, and the B_1_^+^‐based simulation method (C,D) uses only the B_1_^+^ component from simulations. Note that the colormap limits for SAR and 10‐g SAR are different.

The difference between SAR maps retrieved from the simulation‐based and corrected image‐based methods are shown in Figure 9 a. When comparing the simulation‐based map to the image‐based map, the image‐based SAR method yielded lower SAR values in the lateral right region of the brain, and greater values were observed in the posterior lateral left region. The greatest discrepancy was observed around the ventricles. Figure 9 b shows the difference comparison for the two methods for the 10‐g SAR maps. A similar trend was observed to the SAR maps. Similar patterns were observed in SAR (Figure 9 c) and 10‐g SAR (Figure 9 d) maps calculated using the simulated B_1_^+^ field as input. When comparing the simulation‐based map to the image‐based and B_1_^+^‐based maps, lower values were observed in the lateral right region of the brain and greater values in the posterior lateral left region.

Table S1 summarizes the mean [SD] electrical conductivity and SAR values, as well as the range (min–max) for the 10‐g SAR maps for gray matter, white matter, and CSF for the central slice. When comparing the corrected image‐based values with simulation‐based values, higher mean SAR values were observed for the simulation‐based data. However, mean SAR values per tissue were not significantly different (*p* = 0.137).

## 4 DISCUSSION

This work demonstrates the feasibility of obtaining image‐based, subject‐specific SAR maps that present a similar distribution to simulation‐based maps in the brain. Using only clinically available MR sequences, this method introduces a method for analyzing SAR distribution during MRI examinations. The image‐based SAR maps are based on characterizing the B_1_^+^ distribution to retrieve the tissues' electrical conductivity and the E‐field. Specifically, the B_1_^+^ magnitude is obtained via a custom B_1_^+^ sequence,^24^ and the B_1_^+^ phase is acquired with a 2D bSSFP sequence. The proposed image‐based SAR maps were evaluated in vivo on 2 healthy volunteers at 3 T.

Numerical simulations provide high‐resolution SAR mapping, but they require prior knowledge of the RF coil and the patient characteristics. Simulations are also time‐consuming and computationally intensive, limiting their clinical practicality. Consequently, vulnerable patients often undergo MRI without real‐time local SAR evaluation. The proposed image‐based SAR method overcomes this by using only existing MRI sequences that characterize the B_1_^+^ field. A sequence protocol that acquires multislice B_1_^+^ magnitude and phase information in under 9 min was used. Multislice reconstruction of the electrical conductivity, E‐field, SAR, and 10‐g SAR maps took approximately 3 min, demonstrating the feasibility of inline, subject‐specific SAR maps and evaluation during MRI examinations in 12 min.

The proposed method includes subject‐specific electrical properties mapping rapidly derived using the Helmholtz‐EPT method applied to the phase of the B_1_^+^ field with an anatomical filter to mitigate erroneous estimations at the boundaries. In this study, only the electrical conductivity was retrieved via EPT, whereas electrical permittivity was sourced from the literature. Current EPT methods allow accurate characterization of electrical conductivity, although retrieving electrical permittivity via EPT remains an active area of research.^44^ The reconstructed conductivity values using the six‐compartment phantom validation differed from probe measurements by less than 0.04 S/m, providing reliability of the EPT‐based conductivity mapping approach. Similarly, the retrieved electrical conductivity values in vivo were within range of the reference values.^14^, ^41^, ^42^

Several methods have also explored retrieving SAR maps from B_1_ fields.^20^, ^29^, ^30^, ^45^, ^46^, ^47^ To obtain a complete SAR distribution, knowledge of all components of the B_1_ field is necessary. However, because MRI can only characterize the B_1_^+^ distribution, neglecting the B_1_^−^ and B_1z_ components may lead to underestimation of SAR values.^29^ Previous work has suggested using a tissue‐based correction factor to compensate for the SAR underestimation when using only B_1_^+^.^33^ The proposed correction factor was implemented by calculating the ratio of SAR maps derived from the whole E‐field to those derived from the E‐field using only B_1_^+^. In this study, the image‐based SAR maps were corrected using this correction factor. The corrected image‐based SAR maps showed a similar distribution to the simulation‐based SAR maps. The greatest SAR differences were observed at the ventricles. Although slice profile imperfections in the XFL sequence using high flip angles may contribute to these differences,^43^ similar patterns were observed in silico in the present work with smaller flip angles. Boundary errors may also affect the conductivity reconstruction,^48^, ^49^ which can be mitigated using anatomical filters.^50^ In this work, the E‐field calculation used two specialized filters: a standard preprocessing filter for smoothing, followed by an anatomical Savitzky–Golay filter that adapts to tissue boundaries for computing gradients. The standard preprocessing filter, which is not anatomically shaped, may induce cross‐tissue contamination that may not be fully corrected by the subsequent anatomical filter. In regions with sharp transitions, such as the ventricles, these effects could potentially lead to inaccuracies in the E‐field reconstruction and, therefore, in the estimated SAR.

This study also included an estimation of SAR averaged over a 10‐g body volume as recommended by regulatory guidelines.^2^, ^51^, ^52^ Although averaged SAR is not directly linked to temperature increases,^53^ it helps predict worst‐case scenarios and assess safety margins. Postprocessing filtering and data thresholding had a substantial effect on the 10‐g SAR maps, reducing the number of voxels analyzed and potentially removing hotspots at the surface of the brain. Although this issue could be addressed by modifying the 10‐g SAR reconstruction method, comparing different average SAR methods in the obtained maps is beyond the scope of this work. Nonetheless, the image‐based 10‐g SAR maps showed moderate correlation with the simulation‐based 10‐g SAR maps, and the maximum values observed in the 10‐g SAR were consistent between the image‐based and simulation‐based methods.

Other SAR models that use MRI data have also been proposed.^54^ For instance, Gokyar et al. used a 3D neural network that input B_1_ and anatomical maps to derive SAR maps. Although the neural network‐based reconstruction is faster than the imaged‐based SAR method proposed here, it is also prone to errors that underestimate SAR in regions with expected high SAR values. Moreover, the neural network‐based approach did not account for subject‐specific electrical conductivity.

The subject‐specific tissue distribution obtained through EPT remains important for identifying individual anatomical variations, such as tissue volumes and boundaries. This becomes especially relevant for patients whose tissue properties may deviate significantly from standard values due to medical conditions or age.^8^, ^49^, ^55^ Further analysis is necessary to determine how errors in EPT reconstruction affect the accuracy and reliability of SAR estimates.

The precise estimation of local SAR may not be critical for the general patient population at 1.5 T and 3 T. However, subject‐specific SAR assessment could enable optimization of scanning protocols, potentially allowing higher image quality or reduced examination times while maintaining compliance with safety limits established by IEC 60601‐2‐33. Although numerical simulations are the preferred method for local SAR assessment, image‐based SAR maps offer a viable method that can be readily applied in clinical settings for real‐time decision making during MRI exams.

### 4.1 Limitations

This work has some limitations. First, the image‐based SAR approach relied on a skull‐stripping method, which excluded information from the skin and skull. This limits the evaluation to white matter, gray matter, and CSF. Future work is needed to determine whether this method can retrieve SAR information for these surrounding tissues, including a detailed comparison between image‐based and simulation‐based data for external tissues with accurate values.

Second, the proposed method relies on derivatives, which can significantly amplify the B_1_^+^/bSSFP image noise in certain regions. Although an anatomical image‐based filter was used to mitigate noise, it reduced hotspot areas in the SAR and 10‐g SAR maps. The remaining unfiltered noise caused some corrected areas to exhibit higher SAR values than the simulation, although the differences were not statistically significant. More effective filtering and denoising methods are needed to address this issue.

Third, the simulations used as ground truth in this study were based solely on a head model truncated at the neck, which may result in different loading conditions compared with models that include the shoulders or the entire torso.^11^ This truncation could affect the electromagnetic field distributions, as the absence of shoulders and torso alters the RF coil loading conditions. The different loadings might contribute to discrepancies between the simulation‐based and image‐based SAR maps. Additionally, the truncation creates artificial boundaries that affect the path of the induced currents, affecting the electromagnetic field distribution. Additionally, there is a frequency difference between the scanner's operating frequency (123 MHz) and the simulation frequency (128 MHz). Although simulations on a homogeneous cylindrical phantom confirmed minimal differences between these frequencies due to similar electromagnetic wavelengths, further investigation is needed to assess how image‐based SAR maps differ from simulations under various loading conditions or to improve simulation accuracy by integrating the head model with a full‐body model.^11^

Fourth and finally, the comparison between image‐based SAR and simulation‐based SAR assumed identical RF supply conditions, including geometry, position, and input ports. However, even minor variations in these parameters can significantly affect the B_1_^+^ field distribution. A more detailed evaluation of these input values is necessary for a more accurate comparison between image‐based and simulation‐based methods.

### 4.2 Future work

Future work will focus on (i) refining the protocol to retrieve volumetric magnitude and phase B_1_^+^ data with increased SNR and reduced profiling errors, (ii) reducing acquisition time, and (iii) improving filtering and denoising methods during data postprocessing. Further investigation will evaluate the relationship of the preprocessing along with the anatomical filters in the E‐field computation when using only the B_1_^+^ component. Moreover, different methods will be explored to improve the underestimation for tissue boundaries with sharp transitions. An extended analysis will be conducted across various body types and sizes, assessing the feasibility of retrieving maps for patients with medical implants, and analyzing the maps on a wider range of field strengths. This work has primarily focused on MR safety at 3 T. The proposed method has potential applications for generating image‐based SAR maps at higher field strengths, such as 7 T. Additionally, while the correction factors used herein effectively demonstrate the feasibility of image‐based SAR mapping, alternative correction approaches will be investigated to optimize the balance between accuracy and computational efficiency. Finally, there is potential for applying the proposed image‐based SAR method for application in MR‐based RF ablation techniques and for optimizing sequence modifications to achieve subject‐specific B_1_^+^ peak values.

## 5 CONCLUSION

This work demonstrates the feasibility of acquiring image‐based, subject‐specific SAR maps from clinically available sequences. The proposed approach acquires and estimates SAR maps in less than 12 min. Image‐based SAR maps show a distribution like that of simulation‐based SAR maps. Although numerical simulations remain the gold standard for local SAR assessment, when simulations are not feasible, image‐based SAR maps provide a practical alternative that can be easily implemented in clinical practice for real‐time decision making during MRI exams.

## FUNDING INFORMATION

JAM would like to acknowledge support from NIST‐PREP (Professional Research Experience Program), performed under the following financial assistance award 70NANB18H006 from U.S. Department of Commerce, National Institute of Standards and Technology. This paper has been developed based on the results obtained during the 18HLT05 QUIERO Project. This project has received funding from the EMPIR Programme, co‐financed by the Participating States and from the European Union's Horizon 2020 Research and Innovation Programme.

## CONFLICT OF INTEREST

Certain commercial equipment, instruments, software, or materials are identified in this paper in order to specify the experimental procedure adequately. Such identification is not intended to imply recommendation or endorsement by NIST, nor is it intended to imply that the materials or equipment identified are necessarily the best available for the purpose.

## Supporting information


**Table S1.** Mean (standard deviation) electrical conductivity and specific absorption rate (SAR) values, along with the range (min–max) of 10‐g SAR values, for gray matter, white matter, and cerebrospinal fluid (CSF).

## Data Availability

The code used in this study was developed by the authors and is publicly available at: https://github.com/DrJAMM/Image_Based_Subject_Specific_SAR. The datasets generated and/or analyzed during the current study are available from the corresponding author on reasonable request.

## References

[1] Collins CM , Liu W , Wang J , et al. Temperature and SAR calculations for a human head within volume and surface coils at 64 and 300 MHz. J Magn Reson Imaging. 2004;19:650‐656. doi:10.1002/jmri.20041 15112317

[2] International Electrotechnical Commission (IEC). Chapter 201.12 . IEC 60601–2‐33: Medical Electrical Equipment‐Part 2–33: Particular Requirements for the Basic Safety and Essential Performance of Magnetic Resonance Equipment for Medical Diagnosis.

[3] Hecker Prost JE , Wehrli FW , Drayer B , Froelich J , Hearshen D , Plewes D . SAR reduced pulse sequences. Magn Reson Imaging. 1988;6:125‐130. doi:10.1016/0730-725X(88)90441-9 3374283

[4] Murbach M , Zastrow E , Neufeld E , Cabot E , Kainz W , Kuster N . Heating and safety concerns of the radio‐frequency field in MRI. Curr Radiol Rep. 2015;3:45. doi:10.1007/s40134-015-0128-6

[5] Van den Berg CAT , van den Bergen B , Van de Kamer JB , et al. Simultaneous B homogenization and specific absorption rate hotspot suppression using a magnetic resonance phased array transmit coil. Magn Reson Med. 2007;57:577‐586. doi:10.1002/mrm.21149 17326185

[6] Kabil J , Belguerras L , Trattnig S , Pasquier C , Felblinger J , Missoffe A . A review of numerical simulation and analytical modeling for medical devices safety in MRI. Yearb Med Inform. 2016;25:152‐158. doi:10.15265/IY-2016-016 PMC5171558 27830244

[7] Nordbeck P , Fidler F , Weiss I , et al. Spatial distribution of RF‐induced E‐fields and implant heating in MRI. Magn Reson Med. 2008;60:312‐319. doi:10.1002/mrm.21475 18666101

[8] Fiedler TM , Ladd ME , Bitz AK . SAR simulations & safety. Neuroimage. 2018;168:33‐58. doi:10.1016/j.neuroimage.2017.03.035 28336426

[9] Wang C , Shen GX , Yuan J , Qu P , Wu B . Theoretical and experimental investigation of the relationship among SAR, tissues and radio frequencies in MRI. Phys Med. 2005;21:61‐64. doi:10.1016/S1120-1797(05)80020-1 18348846

[10] Homann H , Börnert P , Eggers H , Nehrke K , Dössel O , Graesslin I . Toward individualized SAR models and in vivo validation. Magn Reson Med. 2011;66:1767‐1776. doi:10.1002/mrm.22948 21630346

[11] Wolf S , Diehl D , Gebhardt M , Mallow J , Speck O . SAR simulations for high‐field MRI: how much detail, effort, and accuracy is needed? Magn Reson Med. 2013;69:1157‐1168. doi:10.1002/mrm.24329 22611018

[12] Neufeld E , Lloyd B , Kuster N . From image‐based modeling to the modeling of imaging with the virtual population. In: Tsaftaris SA , Gooya A , Frangi AF , Prince JL , eds. Simulation and Synthesis in Medical Imaging. Springer International Publishing; 2016:45‐54.

[13] Gabriel C . Compilation of the dielectric properties of body tissues at RF and microwave frequencies. 1996. London, UK: King's College London, Dept of Physics. Accessed May 27, 2022. https://apps.dtic.mil/sti/citations/ADA309764

[14] Hasgall PA , Di Gennaro F , Baumgartner C , et al. IT'IS database for thermal and electromagnetic parameters of biological tissues, Version 4.1. Published online February 22, 2022. Accessed August 27, 2024. https://itis.swiss/virtual‐population/tissue‐properties/database/dielectric‐properties

[15] Bottauscio O , Zanovello U , Arduino A , Zilberti L . Polynomial chaos expansion of SAR and temperature increase variability in 3 T MRI due to stochastic input data. Phys Med Biol. 2024;69:125005. doi:10.1088/1361-6560/ad5070 38788726

[16] Villena JF , Polimeridis AG , Eryaman Y , et al. Fast electromagnetic analysis of MRI transmit RF coils based on accelerated integral equation methods. IEEE Trans Biomed Eng. 2016;63:2250‐2261. doi:10.1109/TBME.2016.2521166 26812686

[17] Meliadò EF , Raaijmakers AJE , Sbrizzi A , et al. A deep learning method for image‐based subject‐specific local SAR assessment. Magn Reson Med. 2020;83:695‐711. doi:10.1002/mrm.27948 31483521 PMC6899474

[18] Fraga Rivas P , de Miguel Criado J , del García Salto Lorente L , Gutiérrez Velasco L , Quintana Valcarcel P . Patient safety in magnetic resonance imaging. Radiol Engl Ed. 2023;65:447‐457. doi:10.1016/j.rxeng.2023.01.009 37758335

[19] Santini F , Pansini M , Deligianni X , Caligiuri ME , Oei EHG . ESR essentials: advanced MR safety in vulnerable patients—practice recommendations by the European Society for Magnetic Resonance in Medicine and Biology. Eur Radiol. 2024;35:1785‐1793. doi:10.1007/s00330-024-11055-1 39240349 PMC11913975

[20] Katscher U , Voigt T , Findeklee C , Vernickel P , Nehrke K , Dössel O . Determination of electric conductivity and local SAR via B1 mapping. IEEE Trans Med Imaging. 2009;28:1365‐1374. doi:10.1109/TMI.2009.2015757 19369153

[21] Insko EK , Bolinger L . Mapping of the radiofrequency field. J Magn Reson A. 1993;103:82‐85. doi:10.1006/jmra.1993.1133

[22] Sacolick LI , Wiesinger F , Hancu I , Vogel MW . B1 mapping by Bloch‐Siegert shift. Magn Reson Med. 2010;63:1315‐1322. doi:10.1002/mrm.22357 20432302 PMC2933656

[23] Yarnykh VL . Actual flip‐angle imaging in the pulsed steady state: a method for rapid three‐dimensional mapping of the transmitted radiofrequency field. Magn Reson Med. 2007;57:192‐200. doi:10.1002/mrm.21120 17191242

[24] Amadon A , Cloos MA , Boulant N , Hang MF , Wiggins CJ , Fautz HP . Validation of a very fast B1‐mapping sequence for parallel transmission on a human brain at 7 T. In: *Proceedings of the Annual Meeting of ISMRM*, Melbourne, Australia, 2012. Abstract 3358.

[25] Schmitt T , Rieger JW . Recommendations of choice of head coil and Prescan normalize filter depend on region of interest and task. Front Neurosci. 2021;15:735290. doi:10.3389/fnins.2021.735290 34776844 PMC8585748

[26] Katscher U , van den Berg CAT . Electric properties tomography: biochemical, physical and technical background, evaluation and clinical applications. NMR Biomed. 2017;30:e3729. doi:10.1002/nbm.3729 28543640

[27] Gavazzi S , Mandija S , van den Berg CAT , et al. Sequences for transceive phase mapping: a comparison study and application to conductivity imaging. In: *Proceedings of the Annual Meeting of ISMRM*, Honolulu, Hawaii, USA, 2018. Abstract 5084.

[28] Leijsen R , Brink W , van den Berg C , Webb A , Remis R . Electrical properties tomography: a methodological review. Diagnostics. 2021;11:176. doi:10.3390/diagnostics11020176 33530587 PMC7910937

[29] Voigt T , Homann H , Katscher U , Doessel O . Patient‐individual local SAR determination: in vivo measurements and numerical validation. Magn Reson Med. 2012;68:1117‐1126. doi:10.1002/mrm.23322 22213053

[30] Katscher U , Findeklee C , Voigt T . B1‐based specific energy absorption rate determination for nonquadrature radiofrequency excitation. Magn Reson Med. 2012;68:1911‐1918. doi:10.1002/mrm.24215 22374804

[31] Zhang X , Schmitter S , Van de Moortele PF , Liu J , He B . From complex B(1) mapping to local SAR estimation for human brain MR imaging using multi‐channel transceiver coil at 7T. IEEE Trans Med Imaging. 2013;32:1058‐1067. doi:10.1109/TMI.2013.2251653 23508259 PMC4104985

[32] Zhang X , Van de Moortele PF , Liu J , Schmitter S , He B . Quantitative prediction of radio frequency induced local heating derived from measured magnetic field maps in magnetic resonance imaging: a phantom validation at 7 T. Appl Phys Lett. 2014;105:244101. doi:10.1063/1.4903774 25565707 PMC4272377

[33] Martinez JA , Arduino A , Bottauscio O , Zilberti L . Evaluation and correction of B1+‐based brain subject‐specific SAR maps using electrical properties tomography. IEEE J Electromagn RF Microw Med Biol. 2023;7:168‐175. doi:10.1109/JERM.2023.3236153

[34] Arduino A . EPTlib: an open‐source extensible collection of electric properties tomography techniques. Appl Sci. 2021;11:3237. doi:10.3390/app11073237

[35] Jenkinson M , Beckmann CF , Behrens TEJ , Woolrich MW , Smith SM . FSL. Neuroimage. 2012;62:782‐790. doi:10.1016/j.neuroimage.2011.09.015 21979382

[36] Smith SM , Jenkinson M , Woolrich MW , et al. Advances in functional and structural MR image analysis and implementation as FSL. Neuroimage. 2004;23:S208‐S219. doi:10.1016/j.neuroimage.2004.07.051 15501092

[37] Woolrich MW , Jbabdi S , Patenaude B , et al. Bayesian analysis of neuroimaging data in FSL. Neuroimage. 2009;45:S173‐S186. doi:10.1016/j.neuroimage.2008.10.055 19059349

[38] Adibzadeh F , Paulides MM , van Rhoon GC . SAR thresholds for electromagnetic exposure using functional thermal dose limits. Int J Hyperthermia. 2018;34:1248‐1254. doi:10.1080/02656736.2018.1424945 29347853

[39] The International Commission on Non‐Ionizing Radiation Protection (ICNRP). Guidelines for limiting exposure to electromagnetic fields (100 kHz to 300 GHz). Health Phys. 2020;118:483. doi:10.1097/HP.0000000000001210 32167495

[40] Firbank MJ , Harrison RM , Williams ED , Coulthard A . Quality assurance for MRI: practical experience. Br J Radiol. 2000;73:376‐383. doi:10.1259/bjr.73.868.10844863 10844863

[41] Stogryn A . Equations for calculating the dielectric constant of saline water (correspondence). IEEE Trans Microw Theory Tech. 1971;19:733‐736. doi:10.1109/TMTT.1971.1127617

[42] Martinez J , Arduino A , Bottauscio O , Zilberti L . In silico experimental array of a heterogeneous phantom for EPT reconstruction during temperature increase. In: *Proceedings of the Joint Workshop MR Phase, Magnetic Susceptibility and Electrical Properties Mapping*, Lucia. 2022.

[43] Amadon A , Mauconduit F , Vignaud A , Boulant N . Slice profile corrections in the XFL (magnetization‐prepared turbo‐FLASH) B1‐mapping sequence. In: *Proceedings of the Annual Meeting of ISMRM*, Toronto, Canada, 2015. Abstract 2377.

[44] Mandija S , Meliadò EF , Huttinga NRF , Luijten PR , van den Berg CAT . Opening a new window on MR‐based electrical properties tomography with deep learning. Sci Rep. 2019;9:8895. doi:10.1038/s41598-019-45382-x 31222055 PMC6586684

[45] Cloos MA , Bonmassar G . Towards direct B1 based local SAR estimation. In: *Proceedings of the Annual Meeting of ISMRM*, Honolulu, Hawaii, USA, 2009.

[46] Jiang W , Yang F , Wang K . Individualized and accurate SAR characterization method based on equivalent circuit model for MRI system. Magn Reson Med. 2022;87:2997‐3010. doi:10.1002/mrm.29163 35092069

[47] Balidemaj E , van den Berg CAT , van Lier ALHMW , et al. B1‐based SAR reconstruction using contrast source inversion–electric properties tomography (CSI‐EPT). Med Biol Eng Comput. 2017;55:225‐233. doi:10.1007/s11517-016-1497-6 27108291 PMC5272903

[48] Zhongzheng H , Lefebvre PM , Soullié P , et al. Phantom evaluation of electrical conductivity mapping by MRI: comparison to vector network analyzer measurements and spatial resolution assessment. Magn Reson Med. 2024;91:2374‐2390. doi:10.1002/mrm.30009 38225861

[49] He Z , Soullié P , Lefebvre P , Ambarki K , Felblinger J , Odille F . Changes of in vivo electrical conductivity in the brain and torso related to age, fat fraction and sex using MRI. Sci Rep. 2024;14:16109. doi:10.1038/s41598-024-67014-9 38997324 PMC11245625

[50] Zilberti L , Arduino A , Zanovello U , et al. Magnetic resonance‐based electric properties tomography via Green's integral identity. IEEE Access. 2025;13:42029‐42044. doi:10.1109/ACCESS.2025.3546036

[51] U.S. Food and Drug Administration . Criteria for Significant Risk Investigations of Magnetic Resonance Diagnostic Devices‐Guidance for Industry and Food and Drug Administration Staff. Accessed February 27, 2018. https://www.fda.gov/regulatory‐information/search‐fda‐guidance‐documents/criteria‐significant‐risk‐investigations‐magnetic‐resonance‐diagnostic‐devices‐guidance‐industry‐and

[52] IEC/IEEE International Standard . Determining the peak spatial‐average specific absorption rate (SAR) in the human body from wireless communications devices, 30 MHz to 6 GHz‐Part 1: General requirements for using the finite‐difference time‐domain (FDTD) method for SAR calculations. 62704‐12017. IEC/IEEE, 2017. pp. 1–86.

[53] Wang Z , Lin JC , Mao W , Liu W , Smith MB , Collins CM . SAR and temperature: simulations and comparison to regulatory limits for MRI. J Magn Reson Imaging. 2007;26:437‐441. doi:10.1002/jmri.20977 17654736 PMC4040525

[54] Gokyar S , Zhao C , Ma SJ , Wang DJJ . Deep learning‐based local SAR prediction using B1 maps and structural MRI of the head for parallel transmission at 7 T. Magn Reson Med. 2023;90:2524‐2538. doi:10.1002/mrm.29797 37466040 PMC10543469

[55] Peyman A , Gabriel C , Grant EH , Vermeeren G , Martens L . Variation of the dielectric properties of tissues with age: the effect on the values of SAR in children when exposed to walkie–talkie devices. Phys Med Biol. 2010;55:5249. doi:10.1088/0031-9155/55/17/5249 19088390

