# Supplementary material for: Feasibility study of subject‐specific, brain specific‐absorption‐rate maps retrieved from MRI data
Source: Magn Reson Med. 2025 May 24;94(3):1136–51. doi: 10.1002/mrm.30547 (PMC12202717; doi:10.1002/mrm.30547)
Supplement: Supplementary file 1 — Table S1. Mean (standard deviation) electrical conductivity and specific absorption rate (SAR) values, along with the range (min–max) of 10‐g SAR values, for gray matter, white matter, and cerebrospinal fluid (CSF). [file MRM-94-1136-s001.docx]

|  |  |  | | **Mean [SD]** | | | | **Range (Min – Max)** | | | |  |
| --- | --- | --- | --- | --- | --- | --- | --- | --- | --- | --- | --- | --- |
|  |  | Conductivity [s/m] | | | SAR [W/kg] | | | | 10 g-SAR [W/kg] | | | |
|  |  |  | | | **image-based** | | **Simulation-based** | | **image-based** | | **Simulation-based** | |
|  |  | *Helmholtz-EPT* | *Database* | | *Uncorrected* | *Corrected* |  | | *Uncorrected* | *Corrected* |  | |
| **Gray Matter** | *volunteer 1* | 0.89 [0.35] | 0.59 | | 1.3 [1.6] | 4.1 [4.9] | 3.4 [1.3] | | (0.7 – 2.5) | (1.4 – 5.1) | (1.8 – 5.0) | |
|  | *volunteer 2* | 0.55 [0.22] |  |  | 0.7 [0.4] | 2.3 [1.4] | 3.4 [1.8] | | (0.6 – 2.9) | (1.3 – 6.1) | (0.5 – 6.1) | |
| **White Matter** | *volunteer 1* | 0.49 [0.12] | 0.34 | | 1.5 [1.3] | 2.8 [ 2.2] | 2.6 [1.2] | | (0.7 – 2.2) | (1.4 – 4.7) | (1.6 –5.0) | |
|  | *volunteer 2* | 0.44 [0.16] |  |  | 0.9 [0.5] | 1.6 [0.9] | 2.5 [1.7] | | (0.6 – 2.9) | (1.3 – 5.9) | (0.5 – 5.8) | |
| **CSF** | *volunteer 1* | 1.63 [0.89] | 2.14 | | 1.0 [3.7] | 2.7 [9.7] | 2.7 [1.2] | | (0.7 – 2.2) | (1.4 – 4.5) | (1.9 – 5.0) | |
|  | *volunteer 2* | 1.67 [0.70] |  |  | 0.5 [0.3] | 1.3 [0.8] | 2.9 [2.1] | | (0.6 – 2.9) | (1.3 – 5.6) | (0.5 – 5.2) | |

**Table S1**. Mean [SD] electrical conductivity and SAR values, along with the range (min – max) of 10 g-SAR values, for Gray Matter, White Matter, and CSF.
